# Confronting Covid-19 by exploring the possibility of vaccinating with live SARS-CoV-2 virus itself, via a route that would reduce the incidence of pulmonary complications

**DOI:** 10.12688/f1000research.23480.1

**Published:** 2020-04-29

**Authors:** Etienne Joly

**Affiliations:** 1 Institute of Pharmacology and Structural Biology (IPBS), University of Toulouse, CNRS, Toulouse, 31000, France

**Keywords:** SARS-CoV-2, vaccination, clinical trial

## Abstract

This article proposes that one should explore whether the pulmonary complications of Covid-19 can be reduced or avoided by bypassing the airway entry of the SARS-CoV-2 virus. This could possibly be achieved by injecting live SARS-CoV-2 virus intradermal (ID), subcutaneous, intra-muscular (IM) or intra-peritoneal (IP), or by targeting the virus to the digestive tract.  The effectiveness and innocuity of using those various routes could be tested very rapidly in animal models, such as Macaques, Hamsters, Ferrets or Cats.

The hope is that these experiments will reveal a route of inoculation that can reliably lead to bona-fide infections, resulting in strong immune responses, with both cellular and serological components, but with much less viral replication in the lungs. This would not only hopefully reduce the incidence of pulmonary complications in the infected subjects, but would also probably reduce the amount of virus released by them via aerosols, and thus reduce the vector of contagiosity that is hardest to control, and that probably leads most effectively to viral replication in the lungs.

If those experiments in animal models reveal that one or several routes can be used effectively to reduce pulmonary pathology, a clinical trial could be conducted in human volunteers with very low risk profiles. The ID route should probably be considered as a priority, since it could double-up as a skin test to reveal the immune status of the recipients towards the SARS-CoV-2 virus.

The course of action proposed here may possibly provide a way of taking a step ahead of the virus, and if it works as hoped, could help to end the need for confinement within a matter of months, if not weeks.

## Identifying the real enemy

As of 21 April, over 2 million cases of Covid-19 have been recorded worldwide and thousands are dying every day ( https://ourworldindata.org/coronavirus). It is now clear, however, that the virus only kills a small proportion of those it infects, whilst others recover and become immune in a matter of days. The final outcome of this sanitary war is therefore a foregone conclusion, and the survival of the human race is clearly not at stake.

From the above paragraph, it is clear that the true enemy is not the virus, but time. The true question therefore is: how long will it take to resolve the crisis, and at what human and economic costs?

We thus desperately need to identify rapidly a strategy to reach the only situation that will allow us to put an end to confinement: herd immunity. The approach proposed here consists of exploring if the SARS-CoV-2 virus can be turned into an ally to save time towards reaching suitable levels of herd immunity, and with this the resolution of this unprecedented worldwide crisis. 

SARS-CoV-2 is a highly contagious virus that has two major peculiarities ( Guan *et al*., 2020; Zhu *et al*., 2020):

- The vast majority of serious cases are due to pulmonary complications, following the entry of the virus via the airways and its initial replication in the lungs.
- The morbidity of Covid-19 is massively skewed towards elderly patients, as well as those with pre-existing medical conditions.

It is now clear that the virus causes only mild symptoms in a very significant proportion of the people it infects, and many can even remain completely asymptomatic ( Bendavid *et al*., 2020). Whilst this is fortunate for those asymptomatic carriers, this is also a major factor that must have contributed to the spread of the virus to every single corner of the inhabited world over the course of just a few weeks.

The only way to evaluate what proportions of various populations have been infected by SARS-CoV-2 will be to carry out surveys of the immunological status of large numbers of people representative of those populations. Serological tests are by far the simplest way to carry out such surveys, but the results of those tests can be relatively tricky to interpret because of significant levels of false positives, due to cross-reactivity with other coronaviruses, and false negatives, due to delayed rise in antibody titres ( Okba *et al*., 2020), or to a mostly cellular immune response in certain patients ( Jin *et al*., 2020).

At any rate, the preliminary results of the serological studies carried out on general populations in severely affected areas, where still less than 1% of the population did show clinical signs, seem to suggest that somewhere between 10 and 20% of the population could be already immune to the virus ( Regalado, 2020).

Serological testing will identify people who, having already been infected by SARS-CoV-2 and become immune to it, could come out of confinement and resume a normal life without being a danger to themselves or to others. Several countries are considering issuing sanitary passports to such people. Since there are almost no serious cases in people under 40 years of age with no pre-existing medical condition, if the confinements were to continue for much longer, one could envisage situations in which younger people will start demanding the right to return to a normal life, risking infection by the virus, and thus also risking spreading it to high risk individuals they would interact with. The strategy proposed here represents a possible solution.

Long-lasting confinement will be especially punitive in countries where many people have no source of income unless they go to work. For such people, confinement will rapidly result in starvation, and when hunger hits populations, civil unrest is usually not very far behind. Large numbers of Americans, for example, are employed on “at will” contracts, and have no health benefits. Of greater concern, in several African countries, the confinement imposed by some regimes is predicted to result in the starvation of very large numbers of the poorest people over the coming weeks, due to what has been dubbed ‘Pochvid-20’.

In the strategy proposed here, to speed up the process of developing herd immunity, I suggest taking an approach that would be inconceivable under normal circumstances. Some of the shortcuts suggested here may result in a certain number of casualties. On the other hand, one should not forget that, in the current climate, thousands of people are dying every day, from the virus, in addition to those that are suffering from the consequences of confinement, and that every day saved could thus save as many lives.

## Technical aspects

Immunizing with live viruses has long been recognized as the best way to obtain long-lasting immunity, and the skin is well known as a prime site for immunization, yielding responses that are generally skewed towards a type I T helper response ( Zehrung & Kristensen, 2009). A strong, long-lasting cellular immunity raised against the virus itself rather than against a recombinant construct may be the best way to avoid the manifold problems raised by antibody dependent enhancement (ADE) ( Kulkarni, 2020; Ricke & Malone, 2020; Smatti *et al*., 2018). ADE is one of the major problems that many vaccines developed against lung pathogens have repeatedly encountered over the years. This is one of the reasons why, almost 20 years after the outbreak, we still do not have a vaccine available against SARS-CoV-1, and why a recombinant vaccine against SARS-CoV-2 cannot reasonably be expected to be ready for broad use sooner than 18 months, at the very least ( Ricke & Malone, 2020). On the other hand, the live SARS-CoV-2 virus is the ‘real thing’ and it will have infected well over 2 million people worldwide before the end of this month. Its effects will have been documented better and faster than any other virus before in history, and it is clear by now that lung complications are the primary problem caused by this new virus.

Attenuated viruses have been used to develop very effective vaccines against many of the most lethal viruses, including rabies, yellow fever, polio, measles, mumps, rubella and chicken pox. Some of these vaccines have been found to work after ID inoculation ( Hickling *et al*., 2011; Zehrung & Kristensen, 2009). But obtaining those attenuated viruses took many years of passaging in animals, or in cultured cells, and subsequent testing took at least as long to ensure that the attenuated viruses resulted in suitable immune memory in vaccinated human subjects.

With Covid-19, we do not have this sort of time available, and I would argue that, in the low risk population, the SARS-CoV-2 virus already has the low level of pathogenicity that would make it potentially suitable for a candidate vaccine. Therefore, what I propose to explore is if, by simply changing its route of entry into the organism, one could not turn it into a virus with sufficiently low pathogenicity to be suitable for mass vaccination.

Macaques have already been found to represent a suitable animal model for Covid-19 ( Bao *et al*., 2020; Rockx *et al*., 2020); recent evidence reports that cats and ferrets ( Shi *et al*., 2020) or hamsters ( Chan *et al*., 2020) could also be used, which would open the way for exploring many more avenues in parallel, provided that suitable facilities for performing experiments on those animals can be identified. On the subject of the inoculation route influencing the distribution of the virus, one should highlight that, in a preliminary study, Deng *et al.* found that one of the two macaques inoculated via the eye’s conjunctival route harboured different tissue distributions of viral loads compared to the one macaque inoculated intratracheally ( Deng *et al*., 2020). On this subject, it would be of interest to explore to what extent the different possible routes of contagion by SARS-CoV-2 may contribute to the astounding variability in the intensity of Covid-19 between different patients of similar profiles. Could it be, for example, that lung contagion through aerosols tends to result in more serious clinical outcomes than if the virus is swallowed after passing from hand to mouth?

To bypass the airways, I will argue below that the ID route should be explored as a priority, in one or several of the animal models available if resources permitted it. If the ID route does not result in the efficient triggering of systemic infections, leading to strong immunity and rapid recovery, one could also envisage using subcutaneous, intra-muscular or intra-peritoneal injections, as well as digestive routes, either by ingestion of enterically coated formulations, or via simple intra-rectal (IR) inoculation. If resources permitted, a preferable course of action to save time would be to explore as many of these routes in parallel as possible.

The ID route would, however, seem preferable to the other routes of inoculation, for the following reasons:

- The ID route could also double-up as a skin test, akin to the Mantoux test used to test for BCG-primed immunity. Thus, individuals previously infected by SARS-CoV-2 could be detected in a matter of days. These individuals could then be issued with a ‘sero-converted’ sanitary passport and resume a normal life (and contribute to running the health care systems and to restarting the countries’ economies, without needing to use the protection equipment which is in short supply in so many places).
- It could also boost the immunity of those who have only had mild forms of Covid-19. These people would possibly be at high risk of ADE-related complications in the form of a cytokine storm if they were to be accidentally re-infected by SARS-CoV-2 via the airway route in the course of later months or years, when their level of circulating antibodies would have decreased to sub-neutralizing levels ( Ricke & Malone, 2020; Smatti *et al*., 2018).
- A further advantage of the ID route is that vaccination could be carried out with innocuous material such as micro-needles arrayed on stamps rather than syringes and needles ( Kim *et al*., 2020). And since the virus would only be inoculated into the superficial strata of the skin, the necessary sanitary standards may be more easily attained than for materials destined to be injected with regular needles. Efforts of mass vaccination would therefore be easier to implement.
- To conclude, an advantage of the ID route in my eyes is that with the use of the stamp-like devices carrying micro-needles, volunteers in the early phase trials could self-inoculate the virus, which could circumvent some of the serious ethical issues raised by the approach proposed here.

IR inoculation may turn out to be an alternative to the ID. Indeed, for the IR route, the sanitary considerations would be minimal, and simple dilutions of viral preparations derived directly from tissue culture could conceivably be used. As experience with HIV has shown, some viruses can spread very effectively via the rectal route. Given the fact that ACE-2, the viral receptor, is expressed at very high levels in the digestive tract (see https://www.proteinatlas.org/ENSG00000130234-ACE2/tissue), the virus is expected to replicate efficiently in the gut, and consequently to promote strong mucosal immunity. This is supported by the observation that digestive signs are indeed frequent in Covid-19 patients ( Pan *et al*., 2020), but they do not seem to lead to life threatening complications as frequently as in the lungs ( https://www.sciencemag.org/news/2020/04/how-does-coronavirus-kill-clinicians-trace-ferocious-rampage-through-body-brain-toes#). As an elaboration, one could also conceive of using the IR route as an initial primer, followed by ID inoculation 7 to 10 days later, which would not only boost the immunity, but also provide a direct and sensitive test of the immune status of the recipients.

If the ID or IR routes of inoculation of live SARS-CoV-2 virus were found to cause complications too frequently in the initial stages of the experimentation, a way to work around this undesirable situation may be to use an approach inspired from that used for Polio vaccination nowadays, i.e. the combination of a recombinant live or inactivated vaccine as a first step, followed by inoculation with live virus. Indeed, two different vaccines were initially developed and used against Polio: the inactivated Salk vaccine, which has limited efficacy, but does not have any serious side effects, and the Sabin vaccine, which is using a live attenuated vaccine (the one that is given to kids on a lump of sugar), and provides lifelong immunity. The major drawback of this live vaccine is that it has been found that the attenuated polio virus rapidly reverts to a wild-type, fully pathogenic virus in the intestine of the vaccinated subjects, who will then expel it in very large numbers in their faeces. This is only a very infrequent problem for the vaccinated subjects themselves (in 1/750,000 cases)( John & Vashishtha, 2013), but is of much more concern for the surrounding family members if they are not vaccinated. Nowadays, the recommended course of action for polio vaccination comprises first an immunisation with the inactivated virus, followed a few weeks later by immunisation with the live attenuated virus.

The logistics of such a two-step approach for fighting Covid-19 would, however, be more complicated than the simple inoculation of live virus in a single step. It has been found that the SARS-CoV-2 can easily be grown to very high titres (10 ^7^/ml) in cell lines such as Vero, Caco II, or Calu 3 cells ( Hoffmann *et al*., 2020). With just a few tens of litres of infected cultured cells, one would thus expect to have generated enough live virus to inoculate the whole of the world’s population. But the amount of viral material could become a limiting factor if a pre-immunisation step had to be carried out. Indeed, even if, accounting for the experience acquired with other intra-dermally applied vaccines, another advantage of the ID route is that it would require less material for the inoculation than other routes, such as subcutaneous or intramuscular ( Hickling *et al*., 2011), immunisation with inactivated virus would require several orders of magnitude more material, and thus much more time to produce, than if just live virus was used. And one of the major disadvantages of using inactivated or recombinant vaccines is that, after the vaccination of the first cohort of volunteers, one has to wait to see if the vaccine is indeed protective, which can take a very long time if one waits for the infections to occur naturally. Others have argued that, in order to save time, one should consider inoculating those volunteers with the virus after vaccination ( Eyal *et al*., 2020); this may, however, raise even more ethical issues than the approach proposed herein, especially if one considers the frequent problems of ADE encountered with vaccines against viruses that can cause lung pathologies ( Ricke & Malone, 2020). Personally, given the choice between receiving a live virus via the aerial route after vaccination with a new vaccine or via a route which has been found to seldom lead to lung pathology, I would not hesitate to choose the latter.

The SARS-CoV-2 virus is relatively innocuous for a very large proportion of the population. But a major concern regarding the evolution of this pandemic is that the virus could mutate to become more pathogenic. A further advantage of an approach based on inoculating the virus is that this would completely block any uncontrolled evolution of the virus, as may happen if it is left to spread through populations by simply lifting confinement. It would also mean that serum obtained from any of the immunised recipients could be used to treat those volunteers that would show complications, without any concern for the fact that the virus may be of a sufficiently divergent source that the transferred antibodies would not neutralise the virus efficiently ( Jawhara, 2020).

## The anticipated long-run scenario(s)

The hope is that initial experiments carried out on one or several of the available animal models would find that inoculations with live virus via the ID, IR, or some other route will result in the efficient triggering of systemic infections with SARS-CoV-2 with less pulmonary distribution of the virus than the airway inoculation, and hopefully in faster resolution. If this is indeed found to be the case, one could then envisage to setup a first cohort of a few dozens of human volunteers for inoculation with live virus, via the route(s) that will have been identified as the most promising through animal experimentation. Those volunteers would be selected from populations with very low risks of complications. The trial would also have to take place in a completely confined environment, and under close medical monitoring. I suggest that a military base would be a particularly appropriate environment for this type of project.

Furthermore, it does not seem unreasonable to hope that, by then, some therapeutic approaches will have been identified to combat the virus in patients presenting complications. In this regard, passive immunisation with serum from recovered patients seems to be one of the more hopeful avenues to date ( Jawhara, 2020; Xie *et al*., 2020). In the event that one or more volunteers would show signs of serious complications, it is therefore to be hoped that well-defined therapeutic approaches would be at hand by then to prevent truly serious complications from developing in those volunteers.

In an ideal scenario, the course could be as follows:

1) Over the coming month, completion of the ID inoculation experiments in macaques (with hamsters, ferrets and/or cats as potential additions).
2) During this time, preparations will be made for the first trial on a cohort of a few dozens of volunteers in a completely confined environment. This could take place during the coming month(s), but will obviously only go ahead if and when the results of the above experiments will have shown very promising results.
3) If and when the project gets to that stage of self-inoculating by human volunteers, one of the very important aspects of this experimental stage will be to determine how contagious these inoculated volunteers will become, especially whether they excrete a lot of live infectious virus through their airways, urine, or faeces.
4) If it is found that, after inoculation, the volunteers are not worryingly contagious, one could conceive of an exercise of mass immunisation, strictly on volunteers, asking the inoculated volunteers to stay confined at home during the time they generate immunity to SARS-CoV-2.
5) On the other hand, if it turned out that inoculated individuals can become highly contagious, one may have to resort to performing the immunisations in confined centres, which I refer to as ‘covidodromes’. For this, I envisage that military bases could provide convenient environments, at least for the first stages of the exercise.
6) If it turned out that the approach proposed here was truly efficient, and could result in the rapid sero-conversion of thousands of people with a bare minimum of complications, and even fewer casualties, one could then endeavour to step-up the process to be in a position to offer covidodromes for millions of volunteers to travel to and be inoculated with controlled stocks of virus, in a controlled environment. This could for example be achieved by using tourist places which are currently deserted, such as holiday centres and villages, ski resorts, or even some of the large islands such as those found along the French littoral.

With such a large-scale approach, significant proportions of sero-converted people in the population could be achieved before this summer, ultimately reaching herd immunity, which is the only thing that will allow us to come out of confinement serenely.

## Data availability

No data are associated with this article.
