# Supplementary material for: Confronting Covid-19 by exploring the possibility of vaccinating with live SARS-CoV-2 virus itself, via a route that would reduce the incidence of pulmonary complications
Source: F1000Res. 2020 Apr 29;9:309. [Version 1] doi: 10.12688/f1000research.23480.1 (PMC8108705; doi:10.12688/f1000research.23480.1)
Supplement: Peer review history

# Peer review: Reviewer response for version 1

**DOI:** 10.5256/f1000research.25911.r76622

**Published:** 2021-05-24

**Authors:** Mahmoud Kandeel

**Affiliations:** 1 Department of Biomedical Sciences, College of Veterinary Medicine, King Faisal University, Al‐hofuf, Saudi Arabia

The concept of using life viruses by injection in atypical location might be attractive to some extent and some other researchers had already published on it. However, I am still not encouraged to support this premise as long as safer and effective techniques can be developed. Recently, several vaccines have been approved and widely practiced worldwide, the opinion must be updated.

 The application of live viruses for injection of human being can be a matter of virus activation, mutation and infected or immunized persons might be seeding the virus in the surrounding community and act as a source of infection. This application requires also much care, sophisticated resources and extreme care from the medical staff to avoid contact with the live virus.

 Despite there are some agreements in the literature, there is no solid evidence for the success of this concept. This manuscript also needs to be updated with the recent findings and approvals in the field of COVID-19 vaccines.

 Since the strength of immunity might depend on the amount of virus proteins and poster doses. I am not sure that small amount of live SARS-CoV-2 virus are sufficient to give solid immunity.

Is the topic of the opinion article discussed accurately in the context of the current literature?

No

Are arguments sufficiently supported by evidence from the published literature?

No

Are all factual statements correct and adequately supported by citations?

No

Are the conclusions drawn balanced and justified on the basis of the presented arguments?

No

Reviewer Expertise:

Pharmacology

I confirm that I have read this submission and believe that I have an appropriate level of expertise to state that I do not consider it to be of an acceptable scientific standard, for reasons outlined above.

# Peer review: Reviewer response for version 1

**DOI:** 10.5256/f1000research.25911.r66390

**Published:** 2021-05-10

**Authors:** Jonathan Yewdell

**Affiliations:** 1 National Institute of Allergy and Infectious Diseases (NIAID), Bethesda, MD, USA

Joly proposes immunization with live SARS-CoV-2 (CoV2) via non-respiratory routes as a rapidly employable and cost-effective vaccine. This is a brilliant proposal but compromised somewhat by the introduction of highly effective vaccines for COVID-19. I would recommend that the author update the article accordingly and possibly alter the message to incorporate:

1. CoV2 vaccine effectiveness.
2. Advantages of GI immunization for possibly optimizing mucosal immunity. Possibly mention existing enteric adenovirus vaccines used for US military personnel as a relevant example.
3. Make the obvious point that the proposed approach could be rapidly deployed for emerging CoV2 variants or future pandemic with zoonotic virus.

Is the topic of the opinion article discussed accurately in the context of the current literature?

Yes

Are arguments sufficiently supported by evidence from the published literature?

Yes

Are all factual statements correct and adequately supported by citations?

Yes

Are the conclusions drawn balanced and justified on the basis of the presented arguments?

Yes

Reviewer Expertise:

Adaptive immunity to viruses

I confirm that I have read this submission and believe that I have an appropriate level of expertise to confirm that it is of an acceptable scientific standard.

# Peer review: Reviewer response for version 1

**DOI:** 10.5256/f1000research.25911.r83933

**Published:** 2021-05-05

**Authors:** Ravi Kant Narayan, Manika Verma

**Affiliations:** 1 Etiologically Elusive Disorders Research Network (EEDRN), New Delhi, Delhi, India; 2 Etiologically Elusive Disorders Research Network (EEDRN), New Dehli, Dehli, India

The article proposed the use of unattenuated wild-type SARS-CoV-2 as a live vaccine to be inoculated through routes that can bypass the lungs. Taking into purview the recent trends of the COVID-19 pandemic, the opinion seems to be justified. If we look at the present scenario of the pandemic in India, it would be true to say that the world is going to witness nature's wrath due to human complacency. To avoid further deterioration outside-the-box thinking is required, this article presents the very same. 

 Recent reports have presented facts that differ from those stated in this article. It mentions "Since there are almost no serious cases in people under 40 years of age with no pre-existing medical condition", this can be now refuted by Abbasi (2020) ^1^, which mentions the changing scenario of the pandemic with relation to age distribution.

 To conclude, I would suggest that the opinion being presented should be experimented with in order to search all the avenues.

Is the topic of the opinion article discussed accurately in the context of the current literature?

Yes

Are arguments sufficiently supported by evidence from the published literature?

Partly

Are all factual statements correct and adequately supported by citations?

Partly

Are the conclusions drawn balanced and justified on the basis of the presented arguments?

Partly

Reviewer Expertise:

Anatomy, Genetics

We confirm that we have read this submission and believe that we have an appropriate level of expertise to confirm that it is of an acceptable scientific standard.

## References

[1] : Younger Adults Caught in COVID-19 Crosshairs as Demographics Shift. *JAMA*.2020;324(21) : 10.1001/jama.2020.21913 10.1001/jama.2020.21913 33175133

# Peer review: Reviewer response for version 1

**DOI:** 10.5256/f1000research.25911.r68210

**Published:** 2020-07-29

**Authors:** Ji‐Ming Chen

**Affiliations:** 1 College of Veterinary Medicine, Qingdao Agricultural University, Qingdao, China

This article proposed to use a wild-type SARS-CoV-2 as a live vaccine through intradermal, subcutaneous, intra-muscular, or intraperitoneal inoculation, to bypass the pathogenesis site of the fragile lungs. I published three articles discussing the same opinion (PMID: 32320083 ^1^; PMID: 32525587 ^2^; PMID: 32681650 ^3^). This opinion is supported by the fact that two wild-type adenoviruses have been SAFELY used as live vaccines through enterically coated capsules for 40 years in the US recruits, and reduced over 99% cases of adenovirus-associated acute respiratory diseases (PMID: 27475474 ^4^). My own experiments also suggested that intra-muscular inoculation of a wild-type respiratory virus is safe and able to induce strong immunity (PMID: 32681650 ^3^). 

 With my own experiment, I do believe that it is POSSIBLE and ONLY possible that intradermal, subcutaneous, or intra-muscular inoculation of SARS-CoV-2 is safe and able to induce strong immunity. We should use animal experiments to confirm this route, and such animal experiments are not expensive as compared with the fact that so many people are dying every day and so much economic loss is happening worldwide.

 On the other side, some methods available readily could enhance the safety of the live unattenuated vaccine of COVID-19. For instance, if the SARS-CoV-2 is produced using PK-15 cells rather than Vero cells, it shall carry α1,3-galactose (αGal), and thus can form immune complexes with anti-αGal antibodies which are naturally abundant in human bodies. The anti-αGal antibodies could greatly enhance the safety of the live unattenuated vaccine through blocking the viral entry, aiding complements to destroy the virus, and aiding immune cells to destroy the virus (PMID: 32681650 ^3^). Meanwhile, the anti-αGal antibodies could greatly enhance the efficacy of the live unattenuated vaccine through immune opsonization, as the immune complexes aid the antigen capture, process and presentation of APCs (PMID: 32681650 ^3^). Again, this view should be investigated using animal experiments.

 If the above animal experiments strongly support the opinions, the live unattenuated vaccine of COVID-19 should enter clinical trials in the volunteers. 

 From the view of a veterinary scientist and a long-term worker in public health, I do believe that the live unattenuated vaccine could be much more powerful than the dozens of vaccines currently under rapid development for COVID-19, because the unattenuated vaccine could be of high development speed, high production efficiency, ability to be produced in more  countries, all of which could be as important as the safety of vaccine for reducing deaths and losses of the pandemic.

 In short, I fully support the major opinions of this view: (1) the unattenuated vaccine could be powerful to curb the COVID-19 pandemic; (2) the unattenuated vaccine is imperatively desired to be investigated using animal experiments. Meanwhile, the author should consider the relevant history, publications, and methods to enhance the safety and efficacy of the unattenuated vaccine.

Is the topic of the opinion article discussed accurately in the context of the current literature?

Yes

Are arguments sufficiently supported by evidence from the published literature?

Yes

Are all factual statements correct and adequately supported by citations?

Partly

Are the conclusions drawn balanced and justified on the basis of the presented arguments?

Yes

Reviewer Expertise:

public health; veterinary medicine; control of infectious diseases; epidemiology; influenza; virology; evolution

I confirm that I have read this submission and believe that I have an appropriate level of expertise to confirm that it is of an acceptable scientific standard.

## References

[1] : Decontamination of face masks with steam for mask reuse in fighting the pandemic COVID-19: Experimental supports. *J Med Virol*.2020; 10.1002/jmv.25921 10.1002/jmv.25921 32320083PMC7264583

[2] : Potential for elimination of SAR-CoV-2 through vaccination as inspired by elimination of multiple influenza viruses through natural pandemics or mass vaccination. *J Med Virol*.2020; 10.1002/jmv.26162 10.1002/jmv.26162 32525587PMC7300630

[3] : SARS-CoV-2 replicating in non-primate mammalian cells probably have critical advantages for COVID-19 vaccines due to anti-Gal antibodies: A minireview and proposals. *J Med Virol*.2020; 10.1002/jmv.26312 10.1002/jmv.26312 32681650PMC7404575

[4] : Safety evaluation of adenovirus type 4 and type 7 vaccine live, oral in military recruits. *Vaccine*.2016;34(38) : 10.1016/j.vaccine.2016.07.033 4558-4564 10.1016/j.vaccine.2016.07.033 27475474
